# Supplementary material for: Emergent Magnetic Phenomenon with Unconventional Structure in Epitaxial Manganate Thin Films
Source: Adv Sci (Weinh). 2021 May 6;8(13):2100177. doi: 10.1002/advs.202100177 (PMC8261492; doi:10.1002/advs.202100177)
Supplement: Supplementary file 1 — Supporting Information [file ADVS-8-2100177-s001.pdf]

© 2021 Wiley-VCH GmbH

ADVANCED SCIENCE
Open Access

Supporting Information

for *Adv. Sci.*, DOI: 10.1002/advs.202100177

Emergent Magnetic Phenomenon with Unconventional Structure in Epitaxial Manganate Thin Films

Mingwei Yang, Kuijuan Jin, Hongbao Yao, Qinghua Zhang, Yiru Ji, Lin Gu, Wenning Ren, Jiali Zhao, Jiaou Wang, Er-Jia Guo, Chen Ge, Can Wang, Xiulai Xu, Qiong Wu, and Guozhen Yang*

Supporting Information for

Emergent Magnetic Phenomenon with Unconventional Structure in Epitaxial Manganate Thin Films

Mingwei Yang[1,2], Kuijuan Jin*[,1,2,3], Hongbao Yao[1,2], Qinghua Zhang[1], Yiru Ji[1,2], Lin Gu[1,2], Wenning Ren[1,2], Jiali Zhao[4], Jiaou Wang[4], Er-Jia Guo[1,2,3], Chen Ge[1,2], Can Wang[1,2,3], Xiulai Xu[1,2,3], Qiong Wu[5], and Guozhen Yang[1,2]

[1] Institute of Physics, Chinese Academy of Sciences, Beijing 100190, China

[2] University of Chinese Academy of Sciences, Beijing 100049, China

[3] Songshan Lake Materials Laboratory, Dongguan, Guangdong 523808, China

[4] Beijing Synchrotron Radiation Facility, Institute of High Energy Physics, Chinese Academy of Sciences, Beijing 100039, China

[5] International Center for Quantum Materials, School of Physics, Peking University, Beijing 100871, China

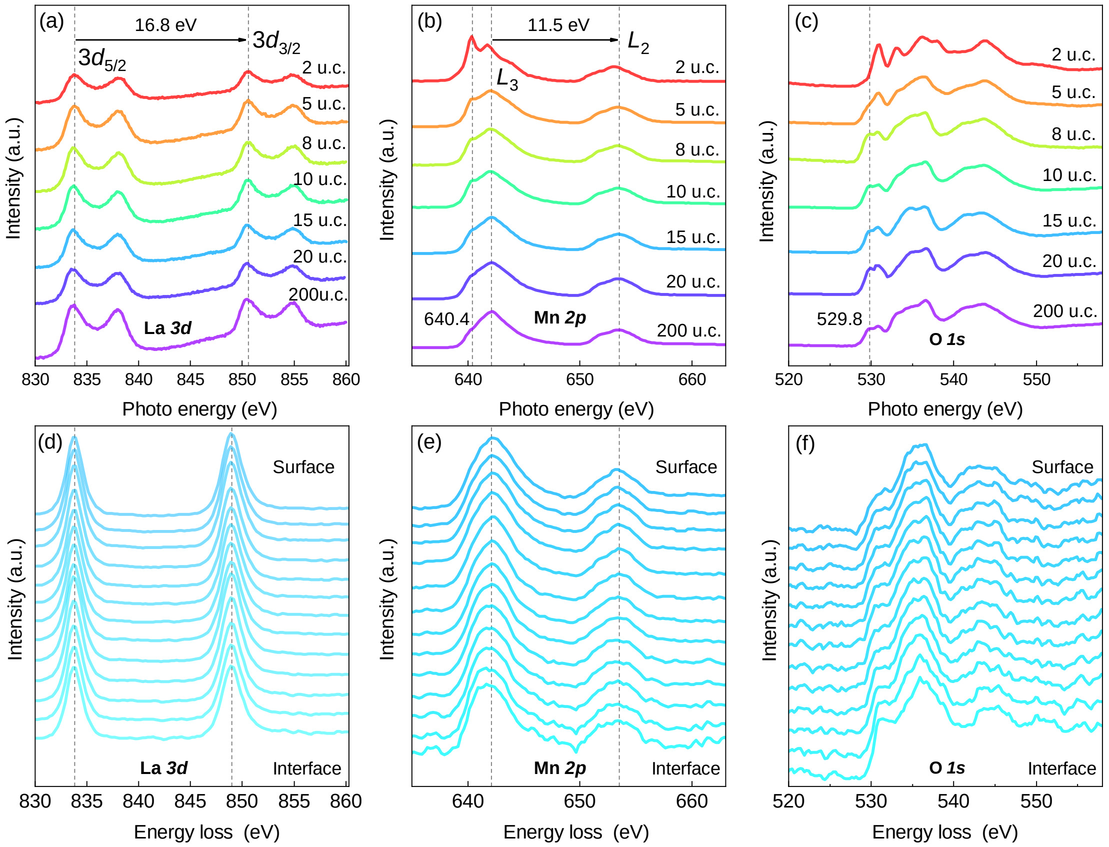

Figure S1. a-c) The x-ray absorption spectroscopy (XAS) spectra for La (*M*-edge), Mn (*L*-edge), and O (*K*-edge) in $LaMnO_3$ (LMO) films, respectively, of 2, 5, 8, 10, 15, 20, and 200 u.c. $LaMnO_3$ (LMO) films. d-f) The unit-cell-resolved electron energy loss spectroscopy (EELS) spectra of La, Mn, and O in a 15 u.c. LMO film. All data have been normalized and offset for better view. The dashed lines represent corresponding peaks indicated by numbers or annotations.

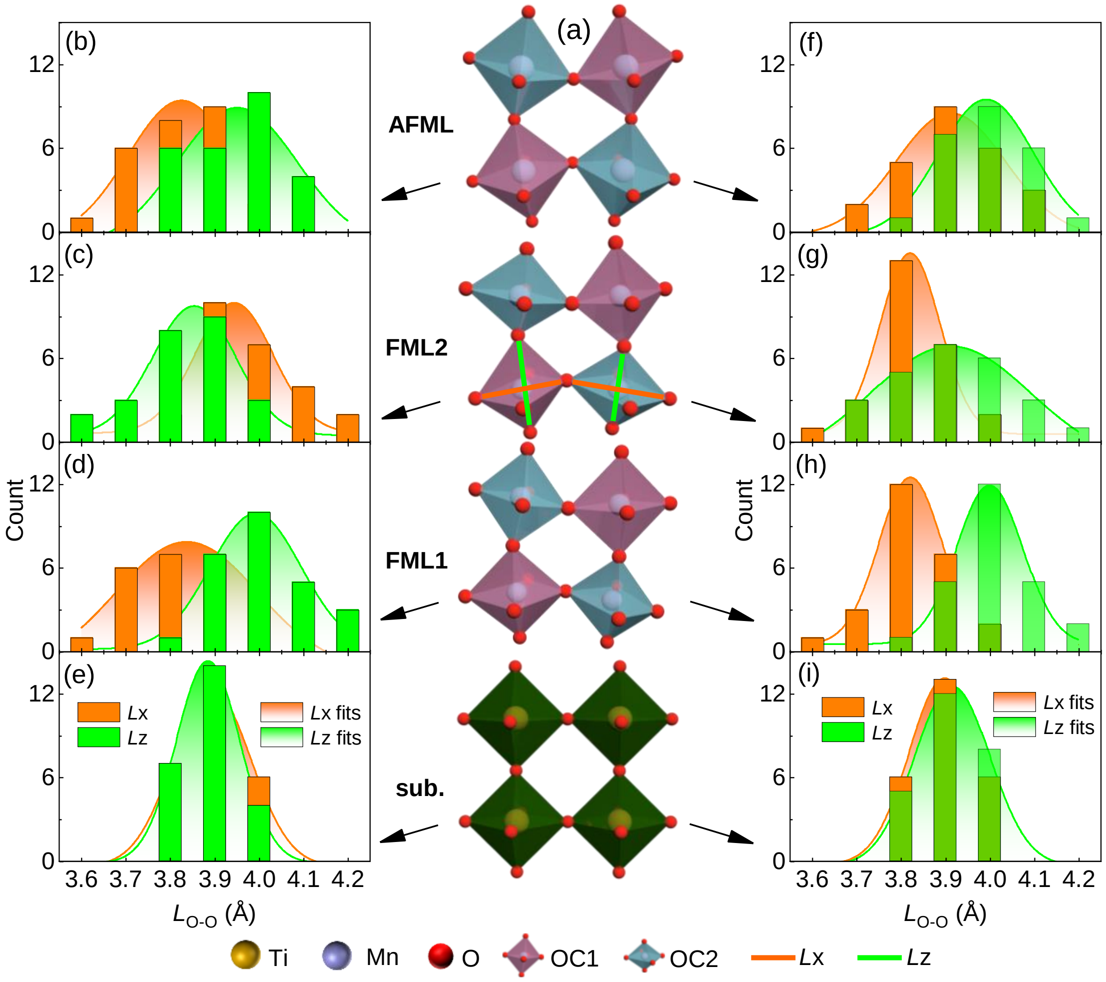

Figure S2. a) Octahedral illustration of the substrate (sub.), first ferromagntic layer (FML1), second ferromagnetic layer (FML2), and antiferromagnetic layer (AFML) referring to Figure 3c. The O-O length ($L_{\text{O-O}}$) in *ab* plane and *c* axis of an octahedron are depicted as *L*x and *L*z, respectively. The brown, mediumslateblue, and red disks denote Ti, Mn, and O atoms, respectively. The purple and dark cyan octahedra, denoted as OC1 and OC2, represent octahedra in two nonequivalent positions, respectively. b-e) Statistical *L*x and *L*z of OC1 in an atomic row (25 columns) in sub., FML1, FML2, and AFML. f-i) Corresponding *L*x and *L*z results of OC2.

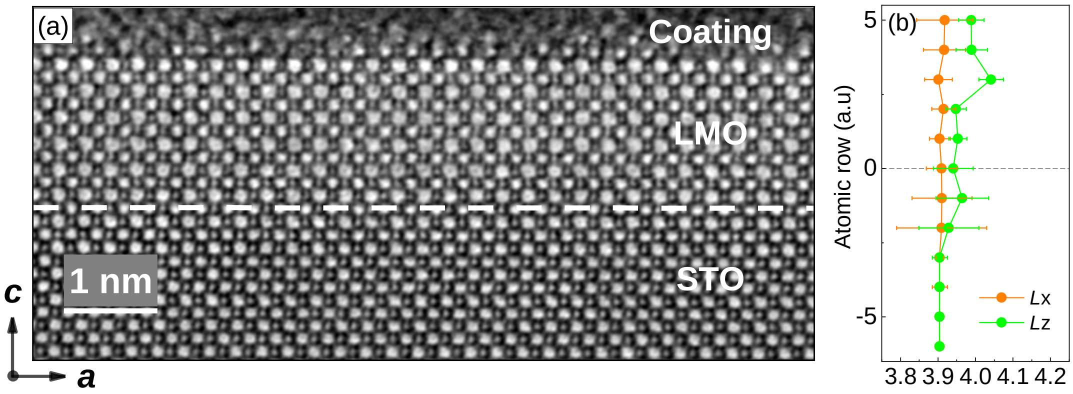

Figure S3. a) Cross section of a 5 u.c. $LaMnO_3$ (LMO) film imaged by scanning transmission electron microscopy (STEM). b) Evaluated O-O length ($L_{O\text{-}O}$) in *ab* plane (*L*x) and *c* axis (*L*z) of octahedra. The dashed lines in a) and b) indicate the sample interface.

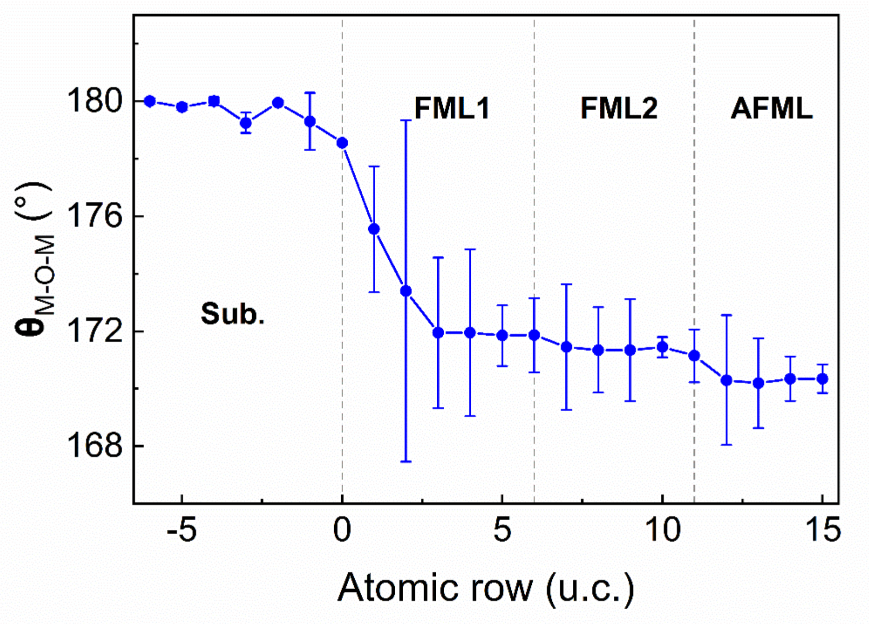

Figure S4. The angle $\theta_{M\text{-}O\text{-}M}$ along *c* axis evaluated from Figure 3e using DigitalMicrograph, where M represents Ti in STO substrate and Mn in LMO layer. The dashed lines indicate the boundaries among the substrate (sub.), first ferromagnetic layer (FML1), second ferromagnetic layer (FML2), and antiferromagnetic layer (AFML). The tilt of octahedra undergoes a monotonous relaxation process from the interface to the surface.

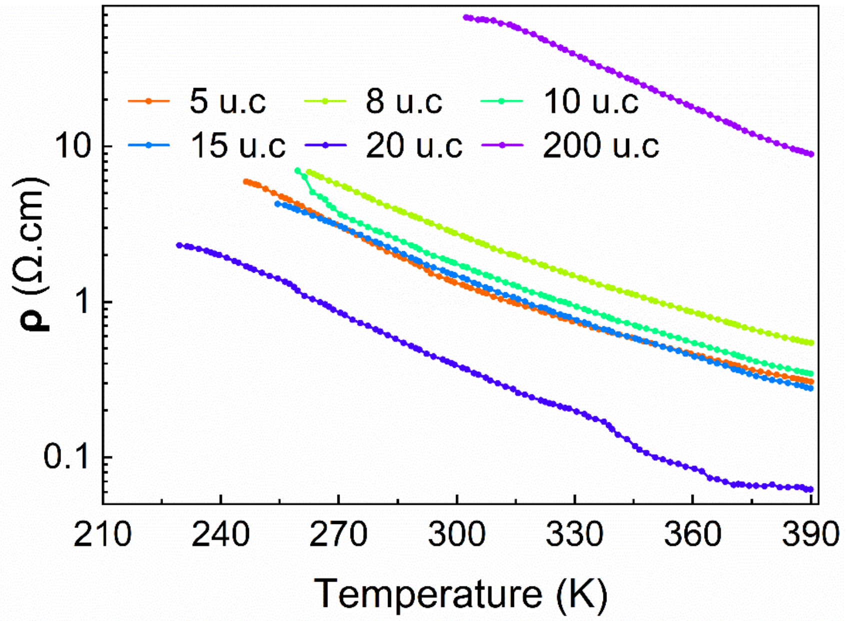

Figure S5. Temperature-dependent resistivity (ρ) of $LaMnO_3$ (LMO) films with thickness of 5, 8, 10, 15, 20, and 200 u.c., respectively.
